# Dual expression and anatomy lines allow simultaneous visualization of gene expression and anatomy

**DOI:** 10.1093/plphys/kiab503

**Published:** 2021-10-29

**Authors:** Britta M C Kümpers, Jingyi Han, John Vaughan-Hirsch, Nicholas Redman, Alexander Ware, Jonathan A Atkinson, Nicola Leftley, George Janes, Giuseppe Castiglione, Paul T Tarr, Kevin Pyke, Ute Voß, Darren M Wells, Anthony Bishopp

**Affiliations:** 1 School of Biosciences, University of Nottingham, Loughborough, LE12 5RD, UK; 2 Howard Hughes Medical Institute, California Institute of Technology, 1200 East California Boulevard, Pasadena, California 91125, USA; 3 Division of Biology and Biological Engineering 156-29, California Institute of Technology, 1200 East California Boulevard, Pasadena, California 91125, USA

## Abstract

Studying the developmental genetics of plant organs requires following gene expression in specific tissues. To facilitate this, we have developed dual expression anatomy lines, which incorporate a red plasma membrane marker alongside a fluorescent reporter for a gene of interest in the same vector. Here, we adapted the GreenGate cloning vectors to create two destination vectors showing strong marking of cell membranes in either the whole root or specifically in the lateral roots. This system can also be used in both embryos and whole seedlings. As proof of concept, we follow both gene expression and anatomy in Arabidopsis (*Arabidopsis thaliana*) during lateral root organogenesis for a period of over 24 h. Coupled with the development of a flow cell and perfusion system, we follow changes in activity of the DII auxin sensor following application of auxin.

## Teaser

A vector system and flow cell set-up allow long-term imaging of both gene expression and anatomy in Arabidopsis primary and lateral roots.

## Introduction

The primary and lateral roots of Arabidopsis (*Arabidopsis thaliana*) provide well-studied systems for cell fate acquisition. Proliferative cell divisions in the root meristem lead to a stereotypic anatomical pattern in which a diarch vascular cylinder is surrounded by radially symmetric layers of outer cells (Dolan et al., 1993). Decades of research into the mechanisms underlying cell fate specification have provided us with a broad set of cell-type specific promoters that can be used to investigate identity of individual files. While we have a good understanding of the mechanistic processes through which a selection of cell identities is established, there are gaps in this knowledge relating to either specific cell types or in understanding how these developmental programs are altered by external stimuli. In addition, anatomical patterning has been well studied in the primary root, but there is much less data over the spatial and temporal control of cell fate specification in the lateral roots. Recent advances in both confocal and light sheet microscopy (Komis et al., 2018; Ovečka et al., 2018) alongside approaches for downstream image analysis have increased our opportunity to record changes in gene activity over long periods of time and follow cell fate specification in emerging organs.

Observing changes in gene expression patterns over time requires both resolving the temporal and spatial dynamics of genes of interest and superimposing these upon the underlying tissue geometry. While genetically encoded fluorescent proteins provide an obvious choice for visualizing either transcription patterns or domains of protein accumulation, there are several options available to resolve the tissue structure. Transmitted light can be used to reconstruct the outline of organs, but it offers little cellular resolution and is unsuitable for creating 3D representations, as it is not possible to obtain z-stacks. The most commonly used alternative is to counterstain with a fluorescent dye such as propidium iodide (PI) or calcofluor white to mark plasma membranes; this approach has become the “go to” standard approach for many scientists. This method is suitable for fixed time points, but suffers a number of drawbacks. First, such stains tend be absorbed strongly within membranes of the outer layers (such as epidermis) and penetrate the innermost layers poorly. In differentiated root tissues, penetration of dyes such as PI is blocked by the endodermis leading to virtually no staining of the stele cells. In fact, exclusion of PI from the stele is used as an assay to test for endodermal differentiation (Alassimone et al., 2010).

Imaging of deep tissues can be improved by fixing and clearing roots using high refractive index mounting media, such as ClearSee (Kurihara et al., 2015) or pseudo-Schiff PI staining (Truernit et al., 2006). These techniques have been modified and used extensively with either fluorescent or β-glucuronidase (GUS) reporters (Truernit et al., 2008; Ursache et al., 2018); however, due to the invasive process of fixation they are unsuitable for live tissues. 

The long-term treatment of Arabidopsis roots with dyes such as PI that is needed for live imaging can introduce problems associated with growth and development. Exposure of cells to extended periods at high levels of PI can render cell membranes vulnerable to permeation. When this occurs, the PI enters the cells and binds to the nucleus preventing its usefulness as a plasma membrane marker. Alternatively, if roots are treated with very low levels the intensity of the PI fades over time, making long-term imaging challenging (Supplemental Figure S1). Genetically encoded fluorescent proteins to mark plasma membranes in a different color to the gene of interest have been used to create a set of different vectors that can be used to co-visualize anatomical structure while following genes of interest, e.g. WAVE lines (Geldner et al., 2009) or fusions between green fluorescent protein (GFP) and the low-temperature-inducible protein, Lti6a or 6b, (Cutler et al., 2000; Grebe et al., 2003; Martinière et al., 2012). However, combining such lines with existing reporter lines requires an extensive crossing program and can delay research by several months.

To facilitate the rapid development of a dual marker system, we incorporated a reporter for a defined gene of interest with a fluorescent plasma-membrane marker with which to observe root anatomy into a single plasmid. Such a system allows long-term imaging of reporters, while reducing the labor required to combine them with genetically encoded membrane markers. The associated red membrane marker is expressed robustly and is bright enough to use as either a selectable marker or proof of transformation for reporter lines where expression may be close to or below the level of detection. We have used this construct mainly for Arabidopsis roots but the membrane marker is also expressed in above-ground tissues. We also developed a variant of this destination vector which outlines cells within the lateral root primordia (LRP) and the stele suitable for long-term imaging of lateral root organogenesis. We have named these destination vectors dual expression anatomy lines (DEALs) and these destination vectors can be easily adapted and customized for any tissue of interest. For long-term imaging and to allow different treatments, we designed a flexible flow cell and perfusion system to maintain healthy roots orientated for imaging for the extended periods made possible by the marker system. This flow cell can be constructed using a 3D printer and used in combination with all standard confocal microscopes. Using this customized flow cell, we followed the expression of our dual markers, observing changes in the dynamics of gene expression following auxin treatment.

## Results

### An efficient method for dual visualization of gene expression and root anatomy

We sought to develop a vector system where we could introduce a single plasmid to plants to simultaneously report gene expression alongside root anatomy. To do this we first tested several different plasma membrane localized fluorophores to identify one with suitable expression in root cells. We selected the pUBQ10::tdTomato 29-1 reporter (Segonzac et al., 2012). This marker gave us consistently good results in all tissues of the primary root of Arabidopsis, although the expression levels were higher in some tissues, such as the root cap.

We have previously used the GreenGate technology for generating plasmid vectors. This is a simple and efficient method that uses the BsaI-type IIS restriction endonuclease to combine six insert modules into a binary destination vector (Lampropoulos et al., 2013). We cloned promoters into the pGGA module for 10 genes that are expressed in a cell type-specific manner and represent most of the common root cell lineages. We also included the constitutively expressed 35S and G1090 promoters, the synthetic cytokinin reporter TCSnew (Pfeiffer et al., 2016) as well as AUXIN TRANSPORTER 1 (AUX1), which is expressed across a variety of tissues in a subset of columella, lateral root cap, and stele cells (see Table  1).

**Table 1:** Promoters used with the DEAL destination vectors

Module name	ATG/ Name	Expressed in	Description	Reference used for primer design/ promoter length
pGGA004 35S	Cauliflower mosaic virus 35S; internal BsaI site removed	Constitutive	GreenGate plasmid	GreenGate plasmid (Lampropoulos et al., 2013)
pGGA pG1090	G‐box10 tetramer fused with CaMV −90/35S promoter	Constitutive	243-bp synthetic sequence	(Ishige et al., 1999)
pGGA pRHD6	AT1G66470 ROOTHAIR DEFECTIVE6 (RHD6)	Epidermis: trichoblasts	2,946-bp upstream of ATG	(Menand et al., 2007)
pGGA	AT1G79840 GLABRA2 (GL2)	Epidermis: atrichoblasts	2,066 bp	(Lee and Schiefelbein, 2002)
pGL2				
pGGA pCO2	AT1G62500 DEG27 CORTEX2 (CO2)	Cortex	568 bp	(Lee et al., 2006)
pGGA pSCR	AT3G54220 SCARECROW (SCR)	Endodermis	2,535 bp	(Gallagher et al., 2004)
pGGA pCRE1	AT2G01830 CYTOKININ RESPONSE1 (CRE1)	Stele	2,094-bp upstream of ATG plus 579-bp downstream of ATG including exon 1 and intron 1	(Mähönen et al., 2006)
PGGA pAUX1	AT2G38120		2,212-bp upstream of ATG	(Swarup et al., 2004)
	AUXIN RESISTANT 1 (AUX1)			
pGGA pAHP6	AT1G80100 ARABIDOPSIS HISTIDINE PHOSPHOTRANSFER PROTEIN6 (AHP6)	Xylem	1,597-bp upstream of ATG	(Mähönen et al., 2006)
pGGA pTMO5	AT3G25710 TARGET OF MONOPTEROS5 (TMO5)	Xylem	2,293-bp upstream of coding sequence	(Schlereth et al., 2010)
pGGA pAPL	AT1G79430 ALTERED PHLOEM (APL)	Phloem	2.9-kb upstream of ATG	(Bonke et al., 2003)
pGGA-pPEAR1	AT2G37590 PHLOEM EARLY DOF1 (PEAR1)	Phloem	1,569-bp upstream of ATG	(Lee et al., 2006)
				(Miyashima et al., 2019)
	previously S9			
pGGA pARR5	AT3G48100 ARABIDOPSIS RESPONSE REGULATOR5 (ARR5)	Procambium	1,611-bp upstream of ATG, 1 BsaI site removed	(D’Agostino et al., 2000)
			1,586-bp promoter fragment (including part of 5′-UTR)	
pGGA043 TCSn	Synthetic cytokinin reporter		339 bp	Plasmid provided by Anne Pfeiffer (Pfeiffer et al., 2016)

**Figure 1:**
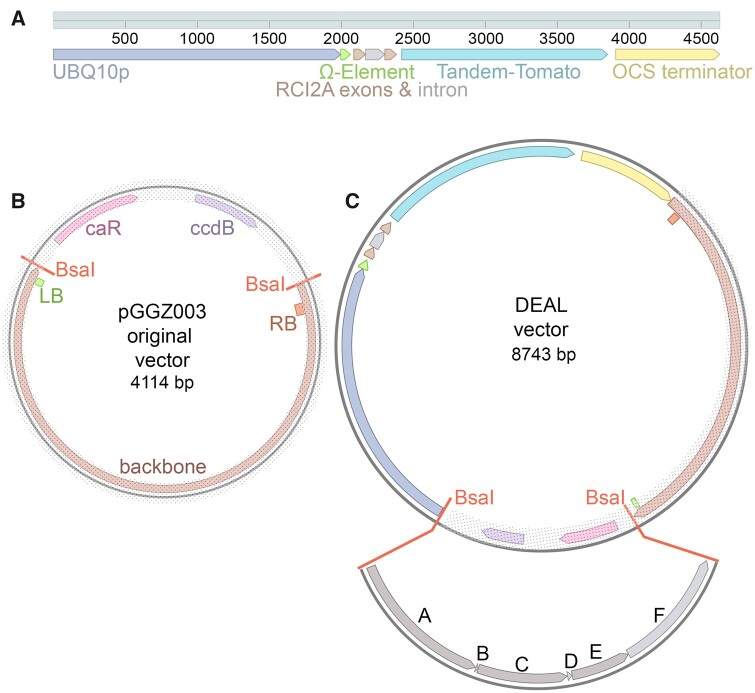
Construction of the DEAL marker system. A, Schematic representation of the UBQ10::tdTomato 29-1 red plasma membrane marker developed by Segonzac et al. (2012) that was introduced into the dual marker system. This consists of a long *UBIQUITIN10* (*UBQ10*, AT4G05320) promoter (2 kb) followed by an Ω element and the *RARE-COLD-INDUCIBLE 2A* gene (*RCI2A*, AT3G05880) coding sequence with intron included fused to tandem tomato (tdTomato), followed by the octopine synthase gene (OCS) terminator. The *UBQ10* promoter drives expression in all cell types and the RCI2A sequence targets the tdTomato to the plasma membrane. B, Schematic representation of the empty pGGZ003 destination vector developed by Lampropoulos et al. (2013). This contains a ccdB cassette and chloramphenicol acetyltransferase gene flanked by two BsaI sites with A and G overhangs, into which GreenGate modules can be cloned. These BsaI sites in turn are flanked by left (LB, shown in green)—and right border (RB, shown in orange) sequences. The backbone requires the presence of the helper plasmid pSOUP in agrobacteria. C, Schematic of the pGGZ003-derived DEAL vector incorporating UBQ10::tdTomato 29-1 next to the RB in the pGGZ003 backbone. For clarity the original pGG003 vector part is shaded. Genes of interest can be inserted into the DEAL vector using the BsaI sites as indicated by the modules shown in gray. Typically, A modules would contain a promoter, B modules an N-tag, C modules a coding sequence, D modules a C-tag, E modules a terminator region, and F modules a resistance marker for expression in planta. Constructs have been visualized using Benchling software (Benchling, 2020).

We tried the methods of using intermediate vectors for double constructs and the oligo duplex method for triple markers as described in the original GreenGate method, but these methods did not work reliably for us. We therefore designed a destination vector including a red membrane vector within the plasmid backbone into which our reporters could be assembled. Our rationale was that any transcriptional/translational reporter could be cloned into this destination vector using a single GreenGate recombination reaction to produce a dual marker highlighting both the gene of interest (e.g. with GFP) and the underlying cell geometry (with tdTomato). For this we used the pGGZ003 destination vector and inserted the DNA sequence encoding the UBQ10::tdTomato 29-1 reporter between the t-DNA right border and the restriction site for the A-module overhang to make our first DEAL marker backbone (Figure  1). This means that the two promoters encoding the red membrane marker and the gene of interest are back-to-back, and therefore read in opposite directions. We chose the pGGZ003 destination vector, rather than pGGZ001, because pGGZ003 has the resistance cassette at the left border, meaning that the resistance cassette is the last part to be inserted into the genome, making it more likely that resistant plants contain the entire constructs.

We used our DEAL destination vector to assemble transcriptional reporters with 14 promoter modules using a standard GreenGate protocol. In each reaction we recombined each A module with the pGGB003 B-module containing a dummy sequence, the pGGC012 C-module containing a nuclear localized GFP or a C-module containing DII-Venus YFP, the pGGD002 D-module containing a dummy sequence, pGGE001 E-module containing the ribulose bisphosphate carboxylase (RBCS) terminator sequence from pea and the pGGF007 F-module encoding Kanamycin resistance. Despite the size of the destination vector increasing from 4,114 bp to 8,743 bp, we did not notice an appreciable change in cloning efficiency using this vector.

**Figure 2:**
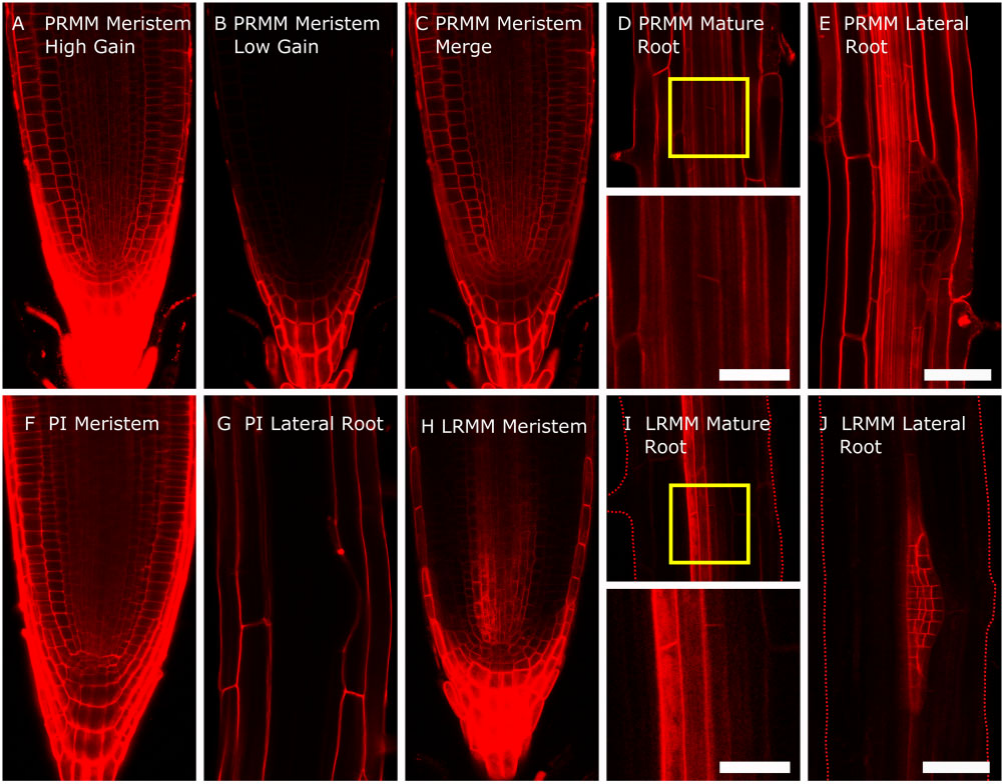
Comparison of the DEAL system with roots counterstained with PI. A–C, Images taken of the root apical meristem of the dual marker system at different confocal settings. A, At high gain settings (500 V) all meristematic cells can be clearly distinguished; however, the older cells in the columella and root cap are saturated. B, At reduced gain (50 V) these cells can be differentiated clearly, although the meristematic cells are barely visible. C, These images can be overlaid using software analysis software such as GNU image manipulation program (GIMP) to give a high-quality composite with every cell being clearly defined. D, Similar to the columella cells, fluorescent intensity is high in the mature tissues. Cell outlines can be clearly defined for all tissues. The yellow box defines a region zoomed in below to showing vascular cell anatomy more clearly. E, Staining in LRP is very weak compared with the overlaying tissues. F and G, Comparable images of control roots counterstained with PI. Note that in the meristem, vascular tissues are not stained as intensely as the dual marker system. In mature roots, PI is unable to enter either the vascular tissues or LRP. H, I, and J, The lateral root specific dual marker system marks cell membranes only in the vasculature, columella, and the LRP. Cells within the LRP are defined clearly, and without fluorescence from the surrounding cells, these primordia can be followed at high resolution. Dashed lines have been used where roots outlines are not readily visible. All images are taken from 7-d-old Arabidopsis plants. The scale bar is 50 μm, except the two magnified images in (D) and (I) where it is 20 μm.

**Figure 3:**
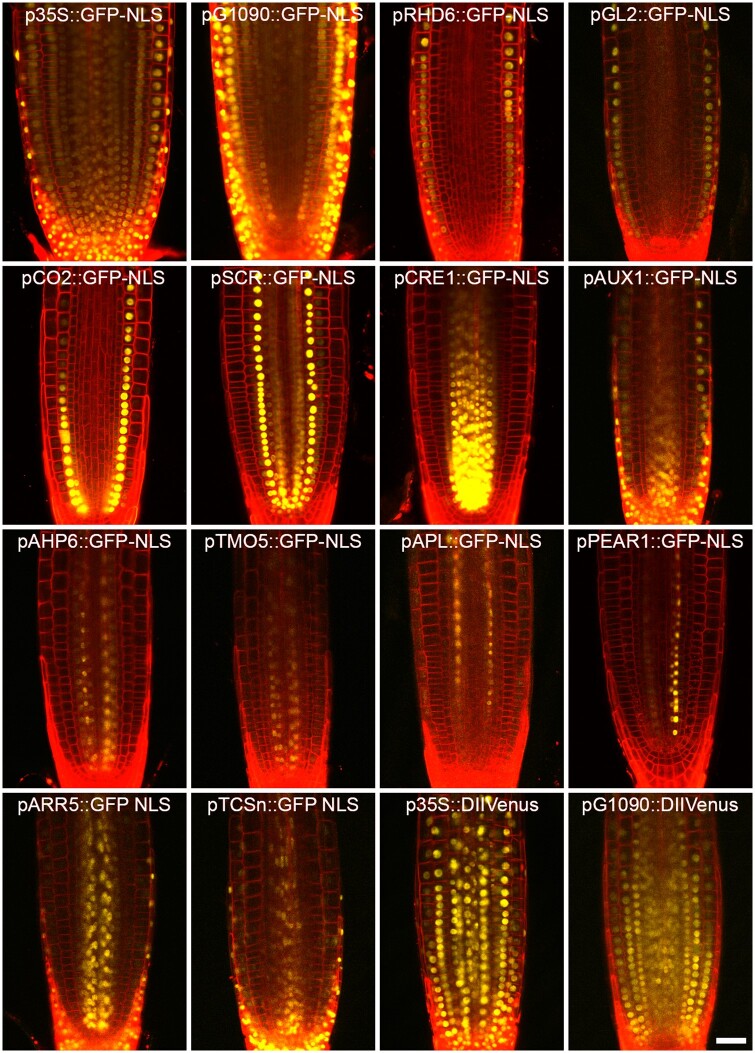
The DEAL constructs allow the identification of major cell types as well as auxin and cytokinin responses. As proof of concept, 16 different constructs were transformed into Arabidopsis covering the following cell types, epidermis, cortex, endodermis, stele, procambium, xylem and phloem, alongside the constitutive promoters 35S and G1090, the cytokinin TCSn reporter and the DII-Venus auxin sensor. Most of these lines contain a nuclear-localized GFP in the C module, except two lines with a DII-Venus module. Primary roots imaged at 7–21 d after germination. Cross sections of the five markers expressed in stele specific cell types (AHP6, TMO5, APL, PEAR1, and ARR5) are provided as movies of *x, z, y* series in Supplemental Movie S1. Scale bar (bottom right): 30 µm.

**Figure 4:**
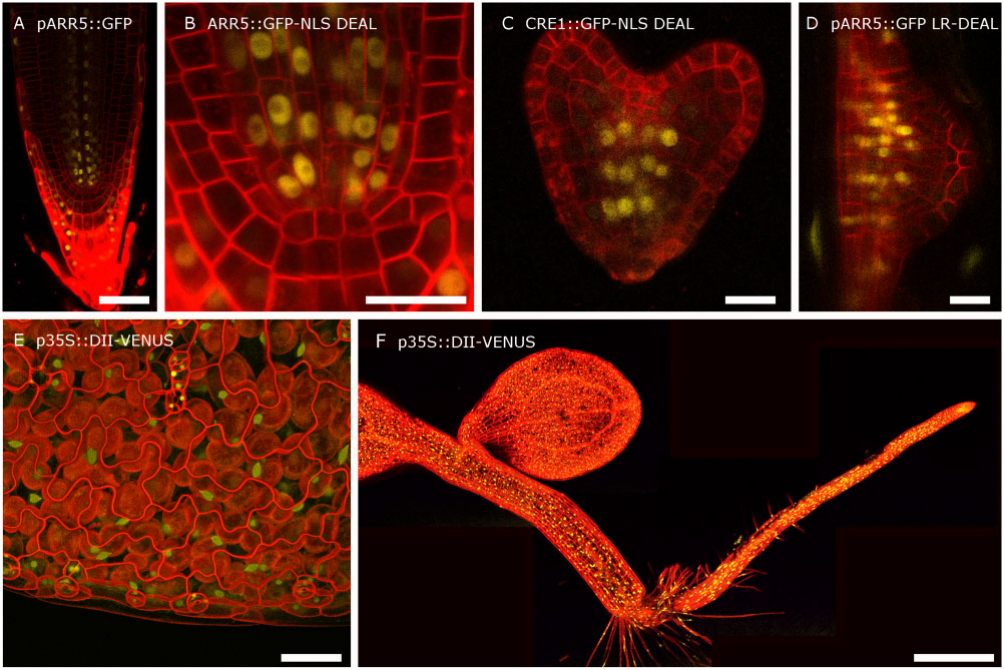
The DEAL system permits imaging in multiple tissues. A and B, An example image of pARR5::GFP-NLS DEAL in the primary root. Part B shows a higher magnification of the stem cell niche. Both images are based on a single exposure with the gain for tdTomato set to 25 and 110, respectively. C, DEAL provides an effective tool for visualizing gene expression in embryos. D, Higher magnification image giving an example of the utility of LR-DEAL during lateral root organogenesis. E, Projection rendered using the ImageJ volume viewer of 35S::DII-VENUS DEAL in cotyledons. This projection was based on a z-series. (F) Reconstruction of an entire 3-d-old seedling of 35S::DII-VENUS DEAL. The image consists of five SD projections stitched together. Scale bars A and E: 50 µm, B, C, and D: 20 µm, and F: 500 µm

These constructs were transformed into Arabidopsis using the floral dip method. There was no appreciable difference in transformation efficiency compared to smaller single marker constructs. These lines allowed us to follow gene expression in the primary root. The DEAL marker shows strong expression throughout the primary root including the vasculature, illustrating that this marker is highly suitable for observing cell anatomy in these tissues (Figures  2, A–D, 3, 4, A and B; Supplemental Movie S1). We observed high fluorescence within the primary root cap. By setting the gain on the confocal detector, we were able to produce good quality images of different tissues despite the differences in fluorescence levels. This caused some imaging complications where cells of different ages were located close to each other, such as in the columella root cap and in LRP (Figure  2, A and E). Here, the older cells had much higher levels of fluorescence. In the case of the root apical meristem, higher gain was required to image the young meristem cells (Figure  2A) while reducing this gain provided optimal exposure for the columella cells (Figure  2B). These two exposures could be merged to render a high-resolution composite (Figure  2C). Using PI as a stain also creates differences in staining intensity, with the outermost cells having the strongest signal (Figure  2F). Overall, the image quality within the meristem zone is comparable to images counterstained with PI. When we look at more mature zones of the root, we can see that the membrane marker outlines the cells throughout the root, including the vascular tissues (Figure  2D), in contrast vascular cells are completely unstained following PI application (Figure  2G). This shows that the DEAL marker is suitable for analysis of all cell types within the main root. Although DEAL was developed for analysis in roots, we also tested whether it was suitable for visualizing gene expression and anatomy in other tissues. We found it to be effective in embryos (Figure  4C) and when imaging cotyledons or even whole seedlings (Figure  4, E and F). When using DEAL in green tissues it is necessary to further gate the wavelength of light collected to prevent the considerable spectral overlap shared between tdTomato and photosynthetic pigments within the plant. One area where the DEAL marker does not excel is in imaging of LRP; Figure  2E). Unlike in PI-stained samples (2G) the cell membranes within the LRP are still marked; however, this is at a much lower level than the adjacent cells. To image LRPs the gain must be set at a level in which both inner and outer layers are overexposed. This will not be a problem for recording images at fixed timepoints but would make the recording of movies challenging. This marker is also expressed in above-ground organs, but this was not further investigated in this project.

### An optimized version for investigating lateral root organogenesis

We wanted to consider how these markers could be applied to investigate changes in gene expression during lateral root organogenesis. In Arabidopsis, the formation of an LRP occurs via a stereotypical series of cell divisions defined by eight stages (Malamy and Benfey, 1997). These stages cover a developmental time series starting by the first asymmetric division in the founder cell through to the establishment of a well-defined primordia. Before the lateral root emerges through the epidermis all major radial cell types are present, although the stage in which vascular cell fates are assigned is less clear. This process from the early stages of division until the establishment of a primordia in which radial cell fate has been defined takes about 24 h (Guseman et al., 2015). As the formation of the lateral root involves the de novo specification of cell fate it provides an excellent template within which to investigate cell-type specification.

**Figure 5:**
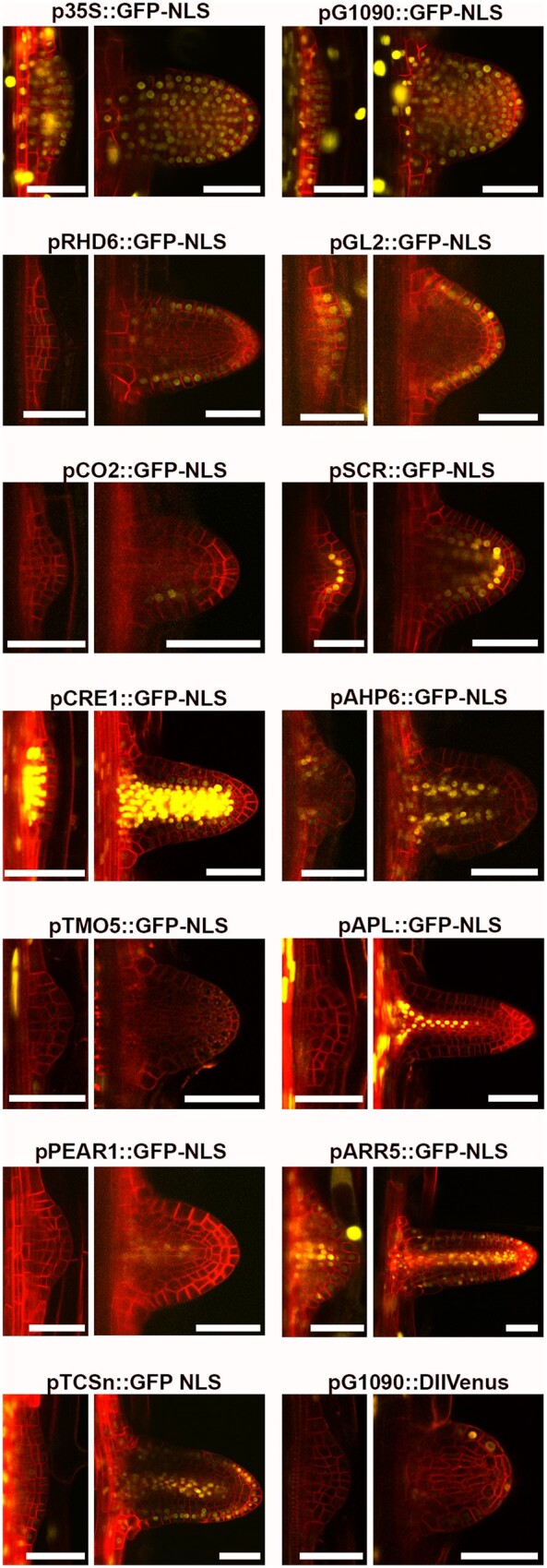
The lateral root specific DEAL constructs allow cell fate and auxin/cytokinin response to be observed in lateral roots of different developmental stages. Fourteen different lateral root specific DEAL constructs to show expression of markers at different stages of lateral root development. For each construct we show the LRP pre-emergence and the emerged lateral root. As for the other DEAL constructs, we used cell-type-specific promoters, the constitutive promoters 35S and G1090 and the cytokinin TCSn reporter and the DII-Venus auxin sensor. In the constitutive promoter lines 35S and G1090, there is also GFP expression in the epidermal cells overlaying the LRP and in the stele. In the pARR5::GFP-NLS lines, there is also some expression visible in the epidermis overlaying the LRP. In several lines (e.g. pRHD6, pPEAR1) there is no GFP expression in the early primordia, but GFP is expressed in later stages. Lateral roots were imaged from 2 weeks after germination. Scale bars: 50 µm.

**Figure 6:**
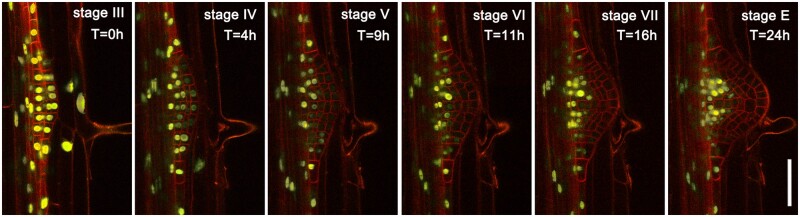
DEAL allow imaging of lateral roots for over 24 h. Time lapse image series of AHP6::GFP-NLS DEAL-LR marker showing progression from stage III through to post emergence. Images were taken every 10 min and represent one plane selected from a *z*-stack. The approximate times and stages of LR development are indicated. See also Supplemental Movie S2. Scale bar: 50 µm.

This process has been followed in real time using either light sheet fluorescence microscopy (Von Wangenheim et al., 2016) or using confocal laser scanning microscopy (Goh et al., 2016). In both cases, genetically encoded plasma membrane markers were used to define plasma membranes. In addition to using the WAVE131Y (Geldner et al., 2009), which expresses a plasma-membrane-localized YFP under a ubiquitous promoter, the study by Goh et al. (2016) also made use of the pAUX1::AUX1-YFP reporter (Swarup et al., 2004), which highlights membranes within the LRP with minimal marking of the overlaying tissue. We first tested our constructs built with the DEAL system at fixed timepoints during lateral root organogenesis to examine their suitability. Although the membrane marker marked the plasma membrane within the LRP, this was not as clear as for the primary root. This is likely because the tdTomato is not turned over quickly and less protein accumulated in the younger cells. If we adjusted the confocal settings for optimum exposure of the LRP, the signal from the overlaying tissues was incredible bright and interfered with imaging. We reasoned that an appropriate solution would be to drive DEAL only within the LRP. Therefore, we designed a second destination vector (LR-DEAL) where we exchanged the UBQ10 promoter with the AUX1 promoter using Gibson assembly. Plants transformed with this vector only had cells within the vasculature, the root tip and the LRPs marked (Figure  2, H–J). As before, we cloned each of our reporters into this vector and followed the expression during lateral root formation (Figure  5). As proof of concept, we applied the confocal imaging approach used by Goh (2016) and took z-stacks of LRP at 10-min intervals. We imaged development from stage III through to post-emergence over a period of 24 h (Figure  6;Supplemental Movie S2). In this time-series, we observed that the AHP6 promoter drove high-level expression within all cells of the primordia at stage II/III, but gradually became restricted to two poles within the vascular tissue as the primordia developed and reached the emergence stage. This demonstrates the utility of our plasmid vectors for uncovering further investigation of gene patterns during lateral root formation, and by producing a series of cell-type-specific markers, we allow future researchers to investigate specification of major cell lineages within the LRP.

### Visualizing dynamic changes in gene expression over time

**Figure 7:**
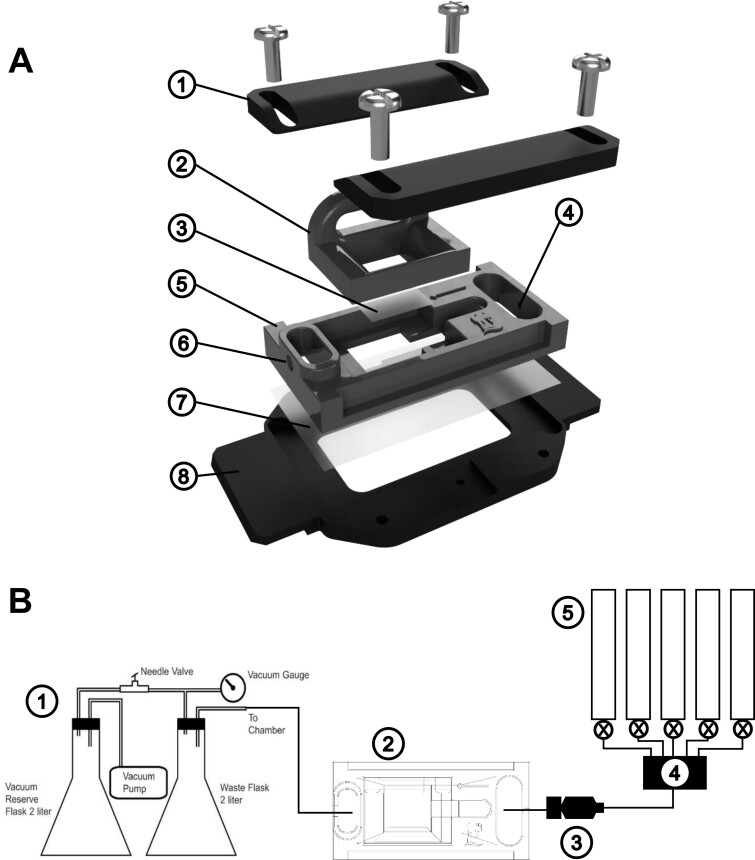
Flow cell and perfusion system. A, Flow cell components. (1) clamps; (2) top support; (3) top coverslip; (4) inlet port; (5) outlet port; (6) flow cell body; (7) cell coverslip; (8) stage adapter. B, Perfusion system. (1) vacuum pump and waste handling; (2) flow cell; (3) flow control valve; (4) manifold; (5) constant volume syringes.

Advances in both microscopy and the development of fluorescent biosensors make live imaging a powerful tool for the study of plant processes at the cellular level. However, keeping plant material alive and orientated to allow imaging during long-term studies is difficult and has resulted in limited adoption of these techniques (Wells et al., 2013; Calder et al., 2015). To allow imaging of roots expressing the DEAL system, we designed a flow cell and perfusion system to maintain healthy roots orientated for long-term confocal microscopy studies. This comprises a 3D-printed flow cell (Figure  7A;Supplemental Figure S2) connected to a constant-pressure perfusion and extraction system (Figure  7B) to allow rapid changes between up to five growth and test media while maintaining the sample root orientated for imaging for prolonged periods on the stage of a confocal laser scanning microscope. The use of 3D-printed components allows flexibility in design of chamber volume and flow characteristics as required. Design and printer files are provided to allow modification of the flow cell to accommodate mounting to different models of inverted microscope. These components can be produced on locally available printers or ordered from one of the many online 3D print services.

**Figure 8:**
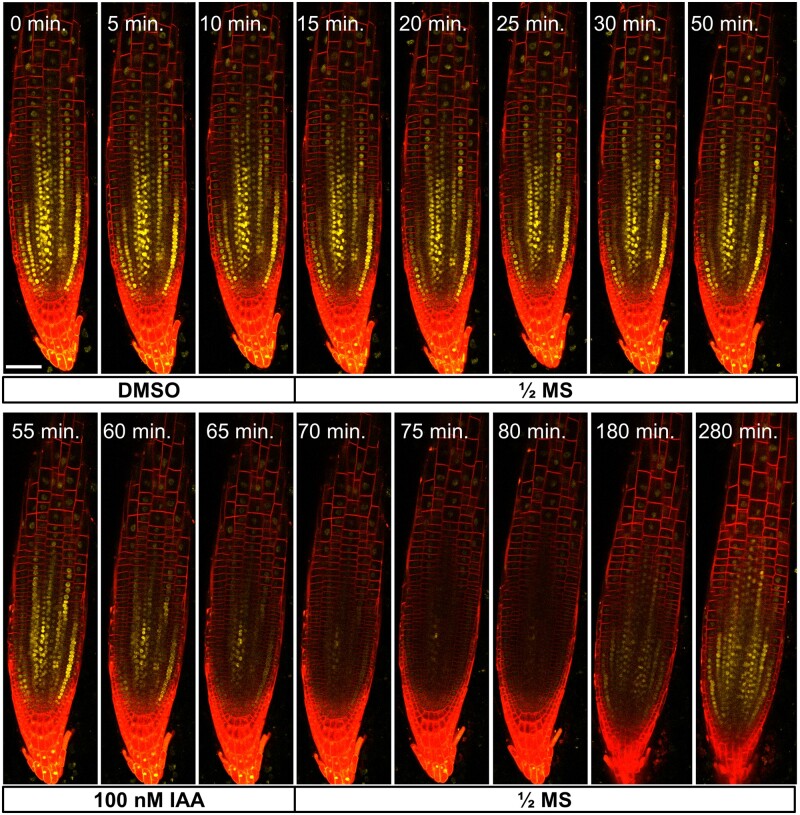
DEAL lines facilitate flow cell imaging without the need for counterstaining. A DEAL line harbouring the auxin reporter line DII-VENUS was imaged using the flow cell and perfusion system over the course of 280 min and subjected to 15-min mock (DMSO) and auxin (100-nM IAA) perfusion treatments at timepoints zero and 55 min. Scale bar: 50 µm. See also Supplemental Movie S3.

The flow cell and perfusion system in combination with the DEAL marker system offers unique advantages for studying root development. Long-term imaging systems such as those developed here are susceptible to the previously outlined issues of cytotoxicity, photobleaching, and subcellular re-localization of cell wall stains, if employed. This can make the assessment of fluorescence levels over time difficult to resolve spatially with regard to their exact cellular position in the root. The red membrane marker vector provides a means to circumvent the above issues, and to obtain positional information for the fluorescence output of a given cell. As a proof-of-concept, we imaged a line harboring the DEAL destination vector with the auxin reporter line DII-VENUS in the flow cell system, subjected to a brief auxin perfusion treatment (15 min, 100-nM indole acetic acid [IAA]) followed by 3 h of recovery, and imaged every 5 min throughout the treatment phases (Figure  8;Supplemental Movie S3). This permitted clear visualization of the degradation and accompanying loss of signal of DII-VENUS in response to the auxin stimulus, with clear resolution of the location of individual nuclei allowing their anatomical position to be called with confidence. For example, it is easy to discern the more rapid loss of DII-VENUS signal in the outer cortical cell layers versus the stele. The DII-VENUS signal was restored ∼3.5 h after removal of the auxin treatment. Furthermore, the membrane marker remained well defined throughout the experiment.

## Discussion

We have developed a comprehensive set of dual expression and anatomical marker lines alongside a flow cell and perfusion system to facilitate long-term live imaging of plants. In addition to providing a suite of marker lines covering most cell lineages in the root, we have developed a GreenGate destination vector that rapidly allows the user to clone their gene of interest into the DEAL system. The two versions of the DEAL system, driven under either the UBQ10 or AUX1 promoter, provide high resolution images in different root tissues. This system can be easily adapted to drive expression of the plasma membrane marker in any organ or cell-type of choice by exchanging the UBQ10/AUX1 promoter with the desired promoter. In this way, it could be utilized for other nonroot tissues. DEAL has several advantages over the conventional method of counterstaining roots with PI; the tdTomato signal is stable over time allowing long-term imaging, particularly in mature roots, we also see vastly improved signal from vascular tissues and by using variants that are expressed in discrete tissues—such as the LR variant—we can focus specifically on the desired structure. When combined with our developed flow cell, these lines provide an efficient way to observe how gene expression or cell identity changes following either environmental stimuli or changes in hormone activity. As proof of concept, we evaluated the response of a DII-Venus line to auxin. Compared with the PI control this provided a vast improvement in methodology, allowing us to observe differences in auxin levels in individual cell types. We were able to follow the process of lateral root organogenesis for over 24 h with no appreciable deterioration in signal.

Whilst the pUBQ10::tdTomato 29-1 or similar membrane marker lines can be introduced into plants individually, incorporating this into a single plasmid offers a significant reduction in experimental time. DEAL also offer a time saving advantage when combined with existing markers by genetic crossing; as both the gene of interest and the anatomical marker are on the same plasmid, they segregate as a single loci. Our labs have now used this system for over 50 transgenic lines and, by using the cloning instructions in the supplemental tables and figures, we can produce high numbers of primary transformants by dipping few plants. We also uncovered a number of advantages in the DEAL system that we did not anticipate. Although we select primary transformants using the conventional antibiotic selection, the red fluorescence is sufficiently high that we can easily screen transgene segregation using a simple fluorescent binocular scope, often before the antibiotic selection becomes apparent (Supplemental Figure S3). We have also found DEAL an ideal method for generating reporters that might show low or even no expression, such as in promoter dissection experiments. In this case the red plasma membrane marker acts as a positive control that the transgene is active.

By providing a suite of pre-made lines, we provide the research community with a toolbox enabling them to pursue projects such as investigating the de novo assignment of cell fate in Arabidopsis lateral roots, or to investigate how hormone responses are modulated by external stimuli. These lines will also be suitable for investigating other adaptive changes such as during biotic or abiotic stresses.

## Materials and methods

### Cloning

#### Greengate entry module construction

Primers with GreenGate overhangs were designed following the Greengate protocol. Control primers were designed at the same time for colony screens and sequencing. For a list of primers see Supplemental Table S1.

#### Construction of destination vectors with red membrane included

The plasmid kit used for generation of plant transformation constructs was a gift from Jan Lohmann (Addgene kit # 1000000036). For our first DEAL destination vector, a red membrane marker construct was added to the original GreenGate pGGZ003 destination vector (Addgene plasmid # 48869; http://n2t.net/addgene:48869; RRID:Addgene_48869) by amplifying the red membrane marker sequence, amplifying the empty destination vector and combining them using Gibson assembly (Gibson et al., 2009). The red membrane marker construct pUBQ10::tdTomato 29-1 (Segonzac et al., 2012) consists of a long UBIQUITIN10 (UBQ10, AT4G05320) promoter (2 kb) followed by an Ω element and the RARE-COLD-INDUCIBLE 2A gene (RCI2A, AT3G05880) coding sequence with intron included (297 bp total) fused to tandem tomato (tdTomato), followed by the OCS terminator at 723 bp in length. For the full sequence, see supplemental tables and figures. The red membrane marker sequence was amplified as a whole from the original construct including the original scar sequences between the different components.

For the lateral root-specific LR-DEAL vector, the first DEAL vector was amplified without the UBQ10 promoter and the promoter was replaced with the AUX1 promoter (AT2G38120, 2,212-kb upstream of ATG) using Gibson assembly. For the primers used in Gibson assembly see Supplemental Table S1.

The assembled DEAL destination vectors were transformed into chemically competent *Escherichia* *coli* DB3.1, which are resistant to the ccdB gene product located in the empty destination vector. The DEAL and LR-DEAL destination vectors were then used in the same way as the original pGGZ003 backbone when assembling GreenGate constructs (Lampropoulos et al., 2013). Destination Vectors will be available through AddGene and plant lines through the Nottingham Arabidopsis Stock Centre.

#### GreenGate assembly

GreenGate assemblies were done using the New England Biolabs Inc. (NEB) GoldenGate mix as this was overall more efficient for us than buying the enzymes separately. All entry modules were checked by sequencing and assembled destination vectors had all seven module borders checked by sequencing to ensure that all modules were present. For a detailed protocol, see supplemental tables and figures.

#### Transformation using floral dipping

Entry modules and destination vectors were multiplied using chemically competent *E. coli* DH5α and were transformed into plants using the floral dip method and electrocompetent *Agrobacterium tumefaciens* (*Agrobacteria*) GV3101 (with pSoup). All destination vectors used in this study had kanamycin resistance *in planta* and were dipped into Col-0 Arabidopsis thaliana plants. T1 seeds were screened on 1/2 murashige and skoog (MS) plates with Kanamycin (50 µg·mL^−1^) and transformants transferred to soil after 2–3 weeks.

### Microscopy

For the primary root image microscopy, T3 plants were grown on 1/2 MS medium supplemented with 1% w/v agar in 16-h light/8-h dark condition for 5 d. For the lateral root primordium image microscopy, T3 plants were grown on 1/2 MS medium supplemented with 1% agar in 16-h light/8-h dark condition for 8 or 10 d. Imaging was performed on either a Leica SP5 or SP8 confocal microscope using sequential scans and either a 40× air or 63× water objective. The tdTomato signal was excited with a 560-nm laser and light between 567 and 701 nm was collected using a Hybrid Detector (Hybrid GaAsp/APD (HyD)). When imaging tdTomato within green tissues (e.g. the cotyledon images in Figure  4) the signal was further restricted to 569–600 nm. Gain values were between 50 and 500 V. For more details see the legend for Figure  2. In the PI comparison figure (Figure  2), roots were stained in 10 µg·mL^−1^ PI for 3 min and washed in distilled water. For the long-term imaging lateral root movie microscopy we used a set up similar to Goh et al. (2016). T3 plants were grown on 1/2 MS medium supplemented with 1% agar and 1% sucrose in 16-h light/8-h dark condition for 8 d. Seedlings were removed from the agar plate and placed in a one-well Nunc Lab-Tek II Chamber Slide with a slice of agar covering the root. Light was supplied externally using a 10-min illumination/1-min dark cycle (in which images were collected) over 24 h. Images were generated with a 63× water objective, and with Zeiss Immersol W. 1.334 used in place of water to prevent evaporation. To speed up data acquisition small z-stacks of only three stacks were taken. Both the tdTomato and GFP were examined with excitation at 488 nm and emission at 496–550 nm for GFP and 590–700 nm for tdTomato.

### Flow cell

The sample holding system consists of a flow cell designed to fit most inverted microscope stages, with dimensions compatible with standard 50 mm × 22 mm glass coverslips (Figure  7A). The flow cell is 3D printed to allow modification to accommodate different size roots and flow characteristics. Components were designed using Fusion 360 CAD software (Autodesk Ltd.) and printed on a stereolithographic 3D printer (Model Form2, FormLabs Inc.) using photocurable resin (Black Tough Resin, FormLabs Inc.). Printer files (*.stl) and original design files (*.f3D) are provided in Supplemental Files and at https://github.com/UoNMakerSpace/flow-cell. Components and suppliers for the system are given in Supplemental Table S2. The flow cell consists of a main body with inlet and outlet perfusion ports and an imaging chamber with a support ramp to position the shoot. A 12 × 12 mm coverslip is fitted to the base of a top support that locates on top of the chamber to control the perfusion volume and prevent excessive movement of the root. A 50 mm × 22 mm coverslip is fitted to the bottom of the main body and sealed with vacuum grease. Once the coverslip has been sealed, the main body of the flow cell is clamped to a stage adapter using two adjustable clamps—this ensures a distortion-free seal for the coverslip. Different stage adapters may be employed for different microscope configurations. To fit Leica microscope stages, we employed a modified P-1 adapter (Warner Instruments, LLC). Before transferring a seedling, a small volume of perfusion solution is pipetted into the imaging chamber. A seedling is then positioned in the chamber with the root in the solution and the stem and cotyledons resting on the support ramp (Supplemental Figure S2). The top support is then fitted, and the chamber filled via the inlet port. This creates a chamber of approximately 27 mm^3^ volume in which a root can be maintained for several days.

Once assembled, the flow cell is moved to the microscope stage and connected to the perfusion and extraction systems (Figure  7B;Supplemental Figure S2). The perfusion system consists of five 60-mL constant flow syringes connected to a five-position manifold with 1.14-mm outer diameter (OD) polyethylene tubing (Harvard Apparatus Inc.). The output of the manifold is connected to the inlet port of the flow cell via a flow valve (Model FR-50S, Harvard Apparatus), allowing fine control (0–10 mL·min^−1^) of solution flow into the cell at a constant pressure head irrespective of the volume of solution in the syringes. The outlet of the flow cell is connected to a vacuum pump with self-contained liquid waste system (Model DWV, Warner Instruments). Balancing the inlet and outlet flows produces a constant flow rate through the root growth chamber. Typically, a rate of 1 mL·min^−1^ is employed, allowing rapid application and removal of test solutions.

For the DII-VENUS experiments with the flow cell, images were taken with a Leica SP8 confocal microscope using a 20×/0.75 dry objective and the Argon 514 nm and HeNe 633 nm laser lines for VENUS and tdTomato excitation respectively. The perfusion media employed was liquid 1/2 MS, pH 5.8 at a flow rate of 1 mL·min^−1^. Once set up in the flow cell, roots were initially perfused with a mock solution (1/2 MS media including 2 µL·L^−1^ DMSO) for 15 min with images taken every 5 min. Imaging continued at 5-min intervals before a 15-min treatment with 1/2 MS media containing 100-nM indole-3-acetic acid (IAA) (in 2 µL·L^−1^ DMSO) at 55 min. Following the IAA treatment, the media was reverted to 1/2 MS and images taken every 5 min to monitor the response until 110 min after which time images were taken every 20–30 min for a further 3.5 h to monitor the recovery of the signal. For the images used to produce Supplemental Movie S3, the red channel was contrast-adjusted using the Enhance Local Contrast plugin (Zuiderveld, 1994) in the FIJI image processing package (Schindelin et al., 2012) to reduce the impact of intensity differences between the root cap and stele.

### Accession numbers

See Table  1 for the list of the accession numbers for major genes/proteins within this article.

## Supplemental data

The following materials are available in the online version of this article.

**Supplemental Table S1.** Primers used to produce entry modules.

**Supplemental Table S2.** Components for the flow cell and perfusion system.

**Supplemental Figure S1.** PI counterstaining does not offer a viable solution for long-term imaging of plants.

**Supplemental Figure S2.** Flow cell mounted on the stage of a Leica SP8 microscope.

**Supplemental Figure S3.** Screening for transformed DEAL seedlings using a dissecting microscope.

**Supplemental Movie S1.** Cross sections of five DEAL constructs with expression in specific vascular cell types.

**Supplemental Movie S2**. Long-term imaging of AHP6::GFP using the LR-DEAL line.

**Supplemental Movie S3**. A DEAL line (red channel) with the auxin reporter line DII-VENUS (yellow channel) was imaged using the flow cell and perfusion system over the course of 280 min and subjected to mock (DMSO) and auxin (100-nM IAA) perfusion treatments for 15 min at timepoints zero and 55 min, respectively.

**Supplemental Files**. Original 3D design files (*.f3D) and printer files (*.stl) are provided for users to modify or print the flow cell. 

## Funding

This work was supported by the Biotechnology and Biological Sciences Research Council [grant numbers BB/L023555/1 (BMCK and AB), BB/M019837/1 (D.M.W. and U.V.) and (BB/M008770/1] (K.P., N.R., J.V.-H.)]; the Royal Society [grant number UF160729 (A.B.)] and the University of Nottingham via the Nottingham Research Fellowship Scheme (U.V.), the Paper Enhancement Fund (A.W., D.M.W., and U.V.) and the Future Food *Beacon of Excellence* (J.A.A. and D.M.W.).

*Conflict of interest statement*. None declared.

## Supplementary Material

kiab503_Supplementary_Data

## Notes

B.M.C.K., J.A.A., D.W., and A.B. designed the concept; B.M.K., J.V.-H. and G.C. built the destination vectors; B.M.K., J.H., A.W., J.V.-H., and A.B. imaged the lines; N.R., J.A.A., and D.W. designed and built the flow cell; G.J., P.T.T., and N.L. donated material or provided entry clones used within this project; K.P. and U.V. supervised parts of this project; B.M.C.K., D.M.W., and A.B. wrote the manuscript with input from all the authors.

The author responsible for distribution of materials integral to the findings presented in this article in accordance with the policy described in the Instructions for Authors (https://academic.oup.com/plphys/pages/general-instructions) is: Anthony Bishopp (anthony.bishopp@nottingham.ac.uk).
