# Supplementary material for: Dual expression and anatomy lines allow simultaneous visualization of gene expression and anatomy
Source: Plant Physiol. 2021 Oct 29;188(1):56–69. doi: 10.1093/plphys/kiab503 (PMC8774739; doi:10.1093/plphys/kiab503)
Supplement: kiab503_Supplementary_Data [file kiab503_supplementary_data.zip › kiab503-suppl_data/PP2021BT00586R2_Supplemental_Tables_Figures_and_Movie_legends.pdf]

Supplemental Files for:
Dual Expression Anatomy Lines (DEAL) allow simultaneous visualization of gene expression and anatomy in roots.

Britta MC Kümpers, Jingyi Han, John Vaughan-Hirsch, Nicholas Redman, Alexander Ware, Jonathan A Atkinson, Nicola Leftley, George Janes, Giuseppe Castiglione, Paul T. Tarr, Kevin Pyke, Ute Voß, Darren M Wells and Anthony Bishopp

Contents
Supplementary Methods
Supplementary Tables S1 & S2
Supplementary Figures S1 & S2
Supplementary Movie Legends
Supplemental Flow Cell Files

Supplemental Methods
Based on experiences, we suggest this simple step-by-step protocol for the construction of entry and destination modules:

Entry modules:

We amplify entry clones by PCR. Prior to cloning these we purify via gel extraction (GeneJET Gel Extraction Kit, Thermo Fisher Scientific). Digestions with BsaI are performed in a single tube in 50 µl volumes using high fidelity enzymes BsaI-HF and CutSmart buffer (NEB), 100 ng of entry vector and 100 ng of purified PCR product. Reactions are incubated at 37° C for one hour, and then the enzyme is deactivated at 85 °C for 10 minutes. The digestion reaction is cleaned using a QIAquick PCR Purification Kit (Qiagen) with the final elution in 25 µl water. To ligate the entry clone, we add 1 µl T4 DNA Ligase (3 u/µl) from Promega with the supplied buffer and water to a volume of 30 µl and incubate at 37° C for one hour. Following deactivation of the enzyme at 70 °C for 10 minutes, we transform 10 µl of the ligation mix into DH5α via heat shock. Plasmids are confirmed from single colonies using Sanger Sequencing.

Destination vectors with NEB golden gate mix

Assembly of the DEAL backbone with all entry modules can be performed in a single tube in a 20 µl reaction, including the following: 1 µl destination vector (DEAL), 2 µl NEB buffer (10x), 1 µl NEB assembly mix (NEB® Golden Gate Assembly Kit (BsaI-HF®v2)),1 µl of each entry plasmid and water up to 20 µl. We dilute our entry & destination modules to a concentration of 75-100 ng/µl. Reactions are then cycled in a thermocycler as below, and transformed to E. coli. We usually screen 5 different colonies per construct, using primers that amplify a region of the construct that is between 500-2000bp long. Junctions between modules are confirmed by sequencing.

Program for the PCR machine:

30 cycles of	37°C	2 min
	16°C	2 min
	55°C	5 min
	80°C	5 min
	4°C	∞

Supplemental Table S1 Primers used to produce entry modules:

Module	Primer name	Primer sequence
AHP6 promoter	AHP6p_GG_F	AACAGGTCTCTACCTCACGGGGCGCAAAGAAGCATGAC
	AHP6p_GG_R	AACAGGTCTCGTGTTCCACAACGGCACACCCGTCTT
APL promoter	APLp_GG_F	AACAGGTCTCTACCTAGTTATGTTTCAAAATAGCGTTAGAT
	APLp_GG_R	AACAGGTCTCGTGTTTCTCTCTCTCTCTCTCTCTG
ARR5 promoter	ARR5p_GG_F	AACAGGTCTCTACCTTGAGAGGTAAAAACCGAGACGATAC
	ARR5p_GG_R	AACAGGTCTCGTGTTATCAAGAAGAGTAGGATCGTGACTC
AUX1 promoter	AUX1p_GG_F	AACAGGTCTCGACCTGAGAACACTGAGAGGTTTAGC
	AUX1p_GG_R	ACCAGGTCTCGTGTTTTTTTTAGCTTCTAGATCTGAGA
CO2 promoter	CO2p_GG_F	AACAGGTCTCGACCTTAACTCCATTATTTACGACTGTGCCACTCT
	CO2p_GG_R	ACCAGGTCTCGTGTTTATCGTTATTAACTAGGGTTCTTGAAATTTTC
CRE1 promoter (including first intron& exon)	CRE1p_GG_F	AACAGGTCTCTACCTCCTAGATTTTCTCACACACCA
	CRE1p_ex2GG_R	AACAGGTCTCGTGTTCTACAACAATAGAGAACAAAAGAA
PEAR promoter	PEARp_GG_F	AACAGGTCTCAACCTGTTTATGTGTTGCCTAACTCTTGATTATTG
	PEARp_RS_R	AACAGGTCTCCCGAATGAGAGCAAAGATATTCCAAGGCAAATACAC
	PEARp_RS_F	ATCTTTGGTCTCATTCGTCTAAAGAGTTCTATATGTTTTGTTCCAAACTGTC
	PEARp_GG_R	AACAGGTCTCATGTTGGTTATTCTCTTTTGATTTTATTCTTCAAAATTCTTA
GL2 promoter	GL2p_GG_F	AACAGGTCTCGACCTGACCTAAGTTTCCTTCAGTATACG
	GL2p_GG_R	ACCAGGTCTCGTGTTACAAATCCTGTCCCTAGCTAG
G1090 promoter	G1090p_GG_F	AACAGGTCTCGACCTATAGTTTAAACTGAAGGCGGGAAAC
	G1090p_GG_R	ACCAGGTCTCGTGTTGGATCCCAGCGTGTCCTCTC
RHD6 promoter (removing 1 internal BsaI site)	RHD6p_GG_F	AACAGGTCTCGACCTCTCAAAGAGGGACAAGACCAA
	RHD6p_RS_R	ACCAGGTCTCAGACGAAAACATGCTAAAACC
	RHD6p_RS_F	ACCAGGTCTCTCGTCTCTAAACCCAAAGAAT
	RHD6p_GG_R	ACCAGGTCTCGTGTTTAGACACTAATAAGTTTGATAAGTGATT
SCR promoter (removing 1 internal BsaI site)	SCRp_GG_F	AACAGGTCTCGACCTATCGCGTAGATACGACCACCACC
	SCRp_RS_R	TTCTCCGGTCTCTCTCCGGTCAATCCTCT
	SCRp_SR_F	AACAGGTCTCCGGAGAAAGACCGGAGAAAGATGGGA
	SCRp_GG_R	ACCAGGTCTCGTGTTGGATCCGGAGATTGAAGGGTTGTTGG
TCSn promoter	promoter provided by Anne Pfeiffer in July 2016. Pfeiffer et al. 2016	
TMO5 promoter (removing 1 internal BsaI site)	TMO5p_GG_F	AACAGGTCTCTACCTGTTGAACGTCGTGTGGGCT
	TMO5p_RS_R	AACAGGTCTCAGACGAAAAAGACTTTTACTTTTAATCAT
	TMO5p_RS_F	AACAGGTCTCTCGTCTCTGGTCGGTCGACA
	TMO5p_GG_R	AACAGGTCTCGTGTTTTTTTGGTTTTTTTGGTTTTTTAGTTTTTGGG
DII Venus C-module	DIIVenus_GG_F	AACAGGTCTCGACCTAAACAAAAAAGCTCGACCAAAGAAAC
	DIIVenus_GG_R	ACCAGGTCTCGTGTTTTACTCTTCTTCTTGATCAGCTTCT

Gibson assembly primers used to produce destination vectors with added red membrane marker

Backbone	Primer name	Primer sequence
pGGZ003 with UBQ10p-RCI2A-tdTomato	UBQTom_gibson_R	AGCGATCGCACCAGGTACCTTGCATGCCGGTCCTGCTGAG
	UBQTom_gibson_F	TTTGAATTCGGTCTCAAGGTATGCATATGAGTCTAGCTCA
	PGGZ3_UBQTom_BackbR	TGAGCTAGACTCATATGCATACCTTGAGACCGAATTCAAA
	PGGZ3_UBQTom_BackbF	CTCAGCAGGACCGGCATGCAAGGTACCTGGTGCGATCGCT
pGGZ003 with AUX1p-RCI2A-tdTomato	AUX1p_GibF	TTTGAATTCGGTCTCAAGGTGAGAACACTGAGAGGTTTAG
	AUX1p_GibR	TGGTAATTGTTGTAAAAATATTTTTTAGCTTCTAGATCTGAG
	PGGZ3_AUX1Tom_BackbGibF	CAGATCTAGAAGCTAAAAAATATTTTTACAACAATTACCAACAA
	PGGZ3_AUX1Tom_BackbGibR	CTAAACCTCTCAGTGTTCTCACCTTGAGACCGAATTCAAA

Supplemental Table S2. Components for the flow cell and perfusion system

Component	Specifications	Model/Filename	Manufacturer
Stage adapter	Modified platform	P-1, modified	Warner Instruments
Perfusion system	Constant flow syringes (60ml x5)	DN/60M	Harvard Apparatus
Manifold	5 to 1 perfusion manifold	MP-5	Harvard Apparatus
Flow controller	Solution flow valve (0 – 10 ml/min)	FR-50S	Harvard Apparatus
Flow Cell	Resin, 3D printed	UoN	UoN
Perfusion Tubing	1.14 ID x 1.57OD	PE-160	Harvard Apparatus
Vacuum system	Vacuum pump with self-contained liquid waste system	DWV	Warner Instruments

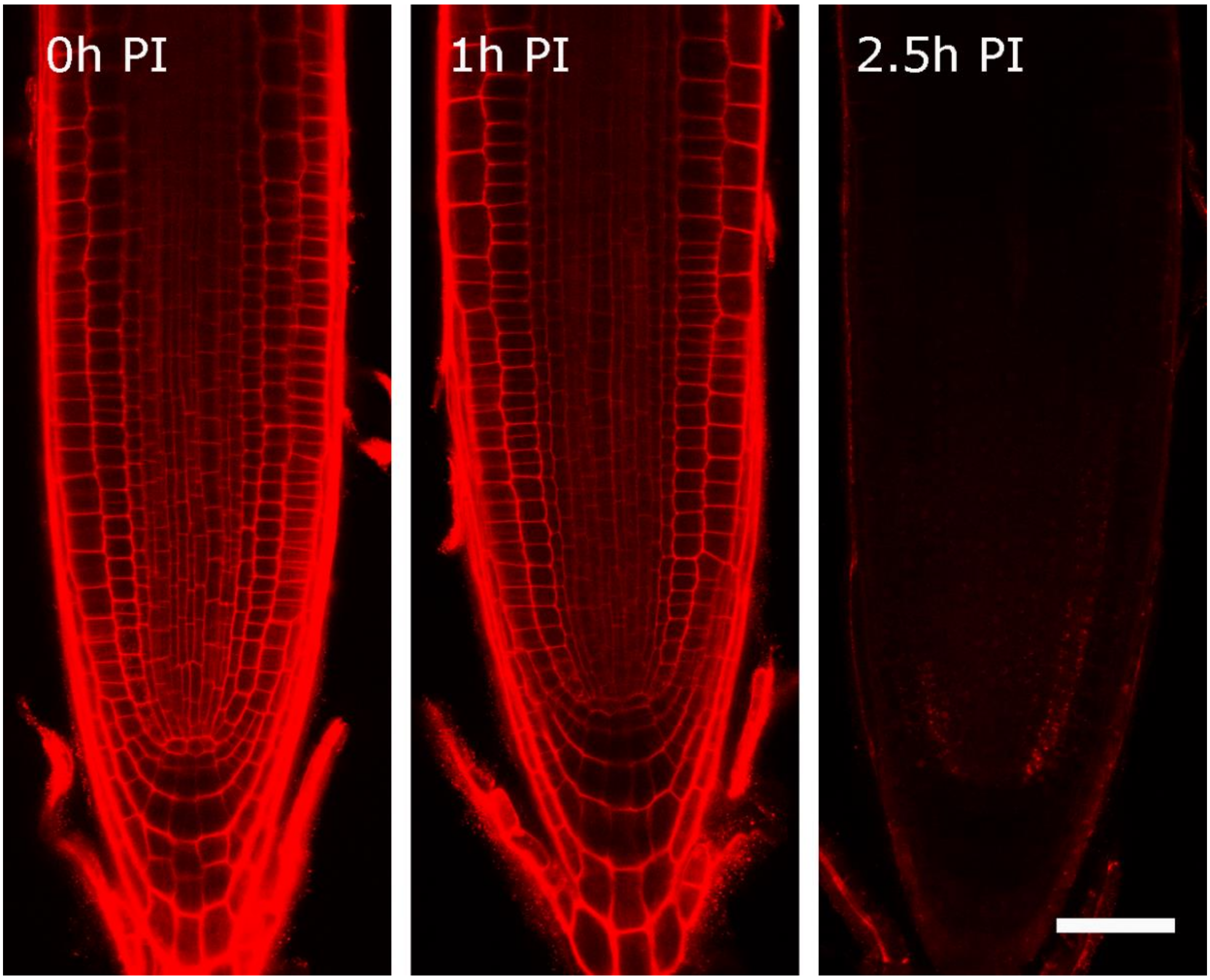

Supplemental Figure S1. Propidium iodide counterstaining does not offer a viable solution for long-term imaging of plants. Time course of Arabidopsis roots stained in propidium iodide. Plants were stained for 3 minutes in 10 µg/ml propidium iodide, rinsed in water and either imaged or placed back on an agar plate. Different individual plants were imaged either directly after washing, 1h later or 2.5h later. Most seedlings could be imaged 1h post treatment with propidium iodide with negligible changes to image quality. However, after 2.5h every plant tested showed very poor staining, with cell walls not being defined and the dye being internalised to intracellular compartments. Representative images are shown. Scale bar: 50 µm.

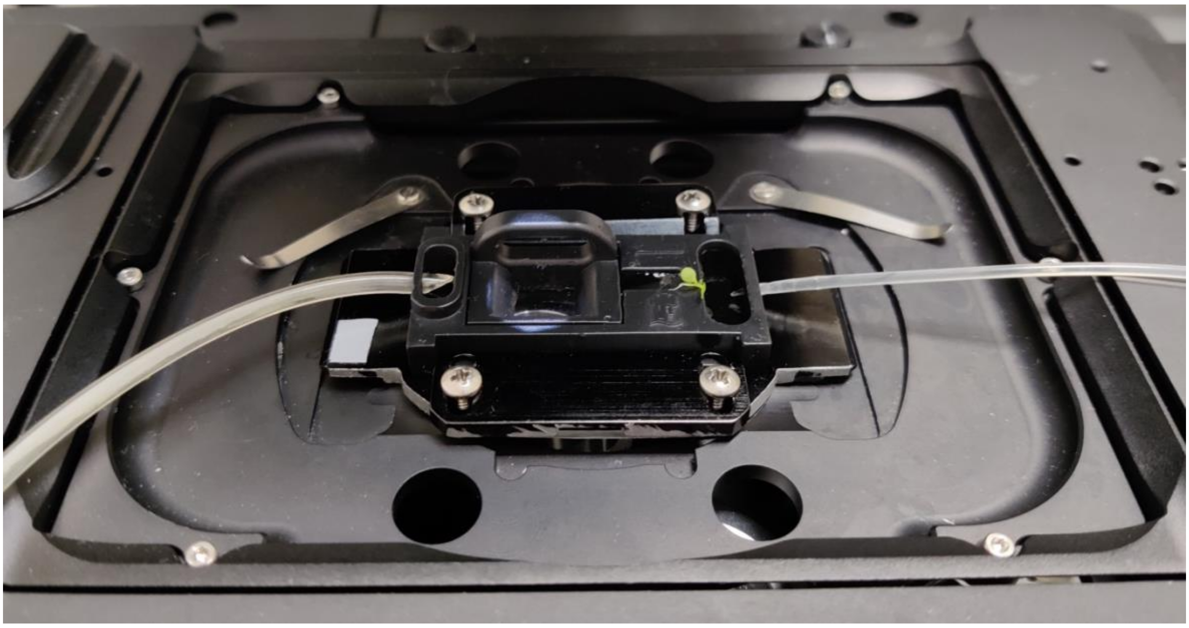

Supplementary Figure S2. Flow cell mounted on the stage of a Leica SP8 microscope.

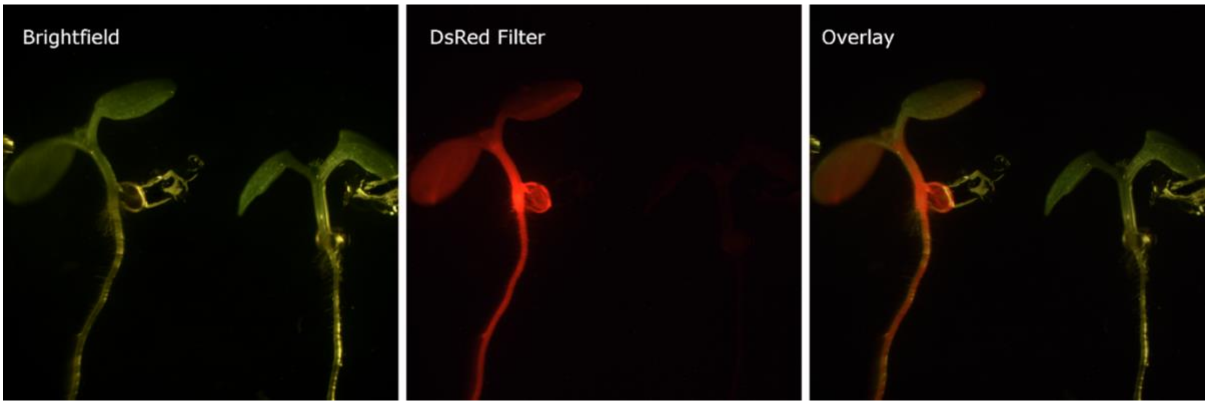

Supplemental Figure S3. Screening for transformed DEAL seedlings using a dissecting microscope. Images of DEAL seedlings taken using a Leica MZ10F stereomicroscope with fluorescent illumination. The example above is taken from a heterozygous line segregating DEAL. Plants were imaged whilst still on an agar plate by turning the plate upside-down, and imaging through the agar and plastic petridish. With our microscope DEAL can be rapidly identified with the DsRed filter cube. We regularly use this approach to identify single loci insertions and to pick homozygous populations of seed. The overlay panel shows the image taken with DsRed overlain the brightfield image with the opacity set at 50%. These images are here to show the ease of screening DEAL lines with a dissecting microscope. Unfortunately, we do not have a camera four our Leica MZ10F microscope and as such we could not obtain high quality images. For that reason we have not included scale bars.

Supplementary Movie Legends

Supplemental Movie S1. Cross sections of five DEAL constructs with expression in specific vascular cell types. It can be difficult to get nuclei in different cells in the same plane, and so for each construct a fly through though a y-series is shown based on an x,z,y projection.

Supplemental Movie S2. Long term imaging of AHP6::GFP using the LR-DEAL line. See also Figure 8. Z-stacks were taken every 10 minutes over a 24h period and a single plane used to assemble the movie.

Supplemental Movie S3. A DEAL line (red channel) with the auxin reporter line DII-VENUS (yellow channel) was imaged using the flow cell and perfusion system over the course of 280 minutes and subjected to mock (DMSO) and auxin (100 nM IAA) perfusion treatments for 15 minutes at timepoints zero and 55 minutes respectively.

Supplemental Files

Original 3D design files (*.f3D) and printer files (*.stl) are provided for users to modify or print the flow cell.
